# Effect of puerarin administration on serum NOD-like receptor protein 3 (NLRP3) inflammasome, C1Q/TNF-related protein 3 (CTRP3) and lipoprotein-associated phospholipase 2 (LP-PLA2) levels

**DOI:** 10.5937/jomb0-53546

**Published:** 2025-06-13

**Authors:** Ge Wang, Qisheng Tang, Dan Wang

**Affiliations:** 1 Beijing University of Chinese Medicine Third Affiliated Hospital, Department of Geriatrics, Beijing, China; 2 Beijing University of Chinese Medicine Third Affiliated Hospital, Beijing, China

**Keywords:** coronary heart disease, puerarin, NLRP3 inflammasome, CTRP3, Lp-PLA2, koronarna bolest srca, puerarin, NLRP3 inflamazom, CTRP3, Lp-PLA2

## Abstract

**Background:**

Coronary heart disease (CHD) is a cardiovascular disease with a high incidence in elderly patients. This article aimed to evaluate the clinical efficacy of puerarin (Pue) as an adjuvant therapy in elderly patients with CHD and the effects of Pue on serum NOD-like receptor protein 3 (NLRP3) inflammasome, C1q/TNF-Related Protein 3 (CTRP3) and lipoprotein-associated phospholipase 2 (Lp-PLA2) levels.

**Methods:**

This retrospective cohort study compared two treatment approaches for CHD patients at Beijing University of Chinese Medicine Third Affiliated Hospital. Fifty-nine patients received standard treatment (5% glucose + nitroglycerin), and 59 received a new treatment (5% glucose + Pue injection). Outcomes were assessed using electrocardiograms, laboratory tests, and cardiac function evaluations.

**Results:**

Relative to the controls, the study subjects had a reduction in the number of angina episodes and an increase in exercise tolerance. In addition, the serum NLRP3 inflammasome and Lp-PLA2 distinctly decreased, and the serum CTRP3 distinctly increased in study subjects.

**Conclusions:**

The administration of puerarin as an adjuvant therapy in elderly patients with coronary heart disease demonstrated significant clinical efficacy, as evidenced by reduced angina episodes and improved exercise tolerance; this might be driven through inflammatory and lipid metabolism pathways.

## Abstract (sr)

**Uvod:**

Koronarna bolest srca (KBS) je kardiovaskularna bolest sa visokom incidencijom kod starijih pacijenata. Ovaj članak je imao za cilj da proceni kliničku efikasnost puerarina (Pue) kao dopunske terapije kod starijih pacijenata sa KBS-om i efekte Pue na serumske nivoe proteina 3 sličnog NOD-receptoru (NLRP3) inflamazoma, proteina 3 povezanog sa C1q/TNF (CTRP3) i lipoproteinske fosfolipaze 2 (Lp-PLA2).

**Metode:**

Ova retrospektivna kohortna studija je uporedila dva pristupa lečenju pacijenata sa KBS-om u Trećoj bolnici Pekinškog univerziteta za tradicionalnu kinesku medicinu. Pedeset devet pacijenata je primilo standardnu terapiju (5% glukoze + nitroglicerin), dok je 59 pacijenata primilo novu terapiju (5% glukoze + injekciju puerarina). Ishodi su procenjeni korišćenjem elektrokardiograma, laboratorijskih testova i procenom srčane funkcije.

**Rezultati:**

U poređenju sa kontrolnom grupom, ispitanici su imali smanjenje broja epizoda angine i povećanje tolerancije na napor. Pored toga, serum NLRP3 inflamazom i Lp-PLA2 su se značajno smanjili, dok se serum CTRP3 značajno povećao kod ispitanika.

**Zaključak:**

Administracija puerarina kao dodatne terapije kod starijih pacijenata sa koronarnom bolešću srca je pokazala značajnu kliničku efikasnost, što je potvrđeno smanjenjem epizoda angine i poboljšanom tolerancijom na napor. To bi moglo biti uzrokovano upalnim i lipid metaboličkim putevima.

## Introduction

Coronary heart disease (CHD) is a common cardiovascular disease, especially in the elderly population. It is caused by lipid-driven chronic immune inflammation and insufficient blood supply to the coronary artery caused by fibroproliferative diseases [1]. It often manifests as chest pain, angina, and other symptoms. It has been reported that the incidence of CHD is still increasing distinctly in developing countries [2]. Due to the decline of physical function and the existence of comorbidities, CHD in elderly patients is often accompanied by more complex clinical manifestations and higher risks [3]. It may lead to severe complications such as myocardial infarction and heart failure [4]. With the aggravation of the ageing trend of the population, the prevention and treatment of CHD in older people has attracted more and more attention. Researchers have further studied the risk factors leading to CHD and carried out the work of primary and secondary prevention in society. In developed countries, the mortality rate has decreased [5]. At present, drug control is mainly used in clinical treatment. Although the short-term effect is good, there are some limitations in conventional Western medicine treatment. Therefore, finding new adjuvant treatment methods has become a current research hotspot.

Puerarin (Pue) is one of the practical components of isoflavones extracted from Pueraria lobata, which has been proven to have various pharmacological effects. It has a protective effect on ischemiareperfusion injury (IRI) of the heart, brain, spinal cord, lung, intestine, and other organs, as well as anti-oxidation, anti-inflammation, and regulation of blood lipids [6] [7]. Its mechanism of action mainly involves regulating multiple signalling pathways and targets, including vascular endothelial cells, inflammatory mediators, and oxidative stress. Pue is therapeutic by inhibiting inflammatory response, reducing myocardial injury, and improving hemodynamics. In the rat model, Zhao et al. [8] found that Pue could down-regulate the expression of NF-κB, enhance the expression of FXR, p-AKT, and p-STAT3, and reduce inflammation, thereby alleviating CHD. Other studies have found that Pue can dilate coronary arteries, increase blood supply, improve heart blood flow, and reduce the attack of angina pectoris. In addition, Pue can inhibit the aggregation of platelets and the formation of thrombosis and reduce the risk of coronary artery occlusion by regulating the factors of the succinate axis through the succinate/HIF-1α/IL-1β axis. In addition, Pue can relieve coronary artery spasms, relax blood vessels, and improve myocardial ischemia and hypoxia [9] [10] [11]. Studies have shown that Pue can effectively prevent the formation of atherosclerosis by reducing the proliferation and migration of hVSMC and the expression of inflammatory factors through the miR-29b-3p/IGF1 pathway [12]. Studies have confirmed that inflammation is related to the development of CHD [13]. IL-6, as a risk biomarker for cardiovascular disease, may have a potential role in atherothrombotic complications, and NOD-like receptor protein 3 (NLRP3) inflammasome is an essential mediator of inflammatory response. Therefore, NLRP3 inflammasome may become a promising target for cardiovascular diseases [14] [15].

C1q/TNF-Related Protein 3 (CTRP3) is a biomarker of coronary artery calcification, and it has been found that the expression of CTRP3 is distinctly reduced in patients with CHD. Moreover, CTRP3 inhibits the osteogenic differentiation of vascular smooth muscle cells induced by high glucose and lipids by inhibiting the nuclear translocation of β-catenin [16]. Some studies have shown that the level of lipoprotein-associated phospholipase 2 (Lp-PLA2) is related to the severity of CHD. The serum level of LP-PLA2 in patients with CHD is distinctly increased, and the level of LP-PLA2 is closely related to the degree of myocardial ischemia and inflammation. LP-PLA2 is a diagnostic marker for CHD [17] [18]. Pue has shown promise in preclinical studies as a potential therapeutic agent for cardiovascular disease. This study aims to investigate the effects of puerarin on specific biomarkers of inflammation and lipid metabolism, which are critical in the pathogenesis of CHD. The primary objective of this study is to investigate the effect of Pue administration on serum levels of NLRP3 inflammasome, CTRP3, and Lp-PLA2 in elderly patients with CHD.

## Materials and methods

### Study design

This study was a retrospective cohort study comparing two groups of patients with CHD who received different treatment approaches.

### Setting and ethical considerations

The study was conducted at Beijing University of Chinese Medicine Third Affiliated Hospital, a primary care hospital in Beijing. The hospital’s Ethics Com mittee approved the study protocol, and all patients provided informed consent.

### Patients

The standard treatment cohort consisted of a historical cohort of 59 patients with CHD who received the standard treatment protocol between 2018 and 2022 before introducing the new treatment approach. This historical cohort served as the control group, allowing us to retrospectively compare the outcomes of patients who received the standard treatment with those who received the new treatment approach.

### Sample size determination

A 1:1 sample size determination method was used to calculate the sample size for this retrospective cohort study. Given the retrospective nature of the study, the sample size was determined based on the available historical data. Since the standard treatment cohort consisted of 59 patients, we aimed to match this sample size for the new treatment cohort to ensure a 1:1 ratio for comparison. Therefore, we planned to recruit 59 patients who received the new treatment approach, allowing for a direct comparison of outcomes between the two groups.

### Treatments

The standard treatment consisted of a 5% glucose injection + nitroglycerin injection 10 mg and an intravenous drip daily for 7 days. This treatment approach was the prevailing treatment protocol at our hospital for several years before the study, and its outcomes were well-documented in our medical records.

From 2023, the study cohort consisted of 59 patients who received the new treatment approach, which was introduced by our specialist team. This treatment consisted of 5% glucose injection 500 mL+Pue injection 0.4 g, intravenous drip once daily for 7 days.

### Outcome measures

We collected data on routine electrocardiograms, blood glucose, blood lipids, and blood routine examinations before and after treatment. We also recorded the frequency of angina pectoris attacks and exercise tolerance (6-minute walk distance, 6MWD) before and after an intervention. Adverse reactions after intervention were also monitored.

### Laboratory measurements

Fasting venous blood (4 mL) was collected from patients before and after administration, and serum was separated and stored at -80°C for later analysis. The serum NLRP3 inflammasome, CTRP3, and Lp-PLA2 levels were measured using enzyme-linked immunosorbent assay (ELISA) kits (human NLRP3 ELISA kit, ab274401; human CTRP3 ELISA kit, CSB-E13426h; human Lp-PLA2 ELISA kit, ab235643).

### Cardiac function assessment

Cardiac function was assessed using echocardiography (Kunlun Resona R9 colour Doppler ultrasound, Mindray) before and after treatment. Left ventricular end-systolic diameter (LVESD), left ventricular end-diastolic diameter (LVEDD), left ventricular ejection fraction (LVEF), cardiac index (CI), and stroke index (SI) were measured and recorded.

The criteria for therapeutic efficacy are displayed in Table 1.

**Table 1:** Evaluation of Efficacy.

Evaluation of efficacy	Angina pectoris attack reduction rate	Resting electrocardiogram	Cardiac insufficiency
Conspicuous effect	80% or higher	Return to normal	Return to normal
Effective	50%; 79% or less	Improvement	Improvement
Ineffective	49% or less	No change or aggravation	No change or aggravation

### Statistical analysis

SPSS24.0 software was employed for processing; measurement data were expressed as mean ± standard deviation (±s), and a *t*-test was used for contrast. Count data were expressed as percentages (%) and compared using χ^2^ tests. *P*<0.05 was considered statistically meaningful.

## Results

**Figure 1:**
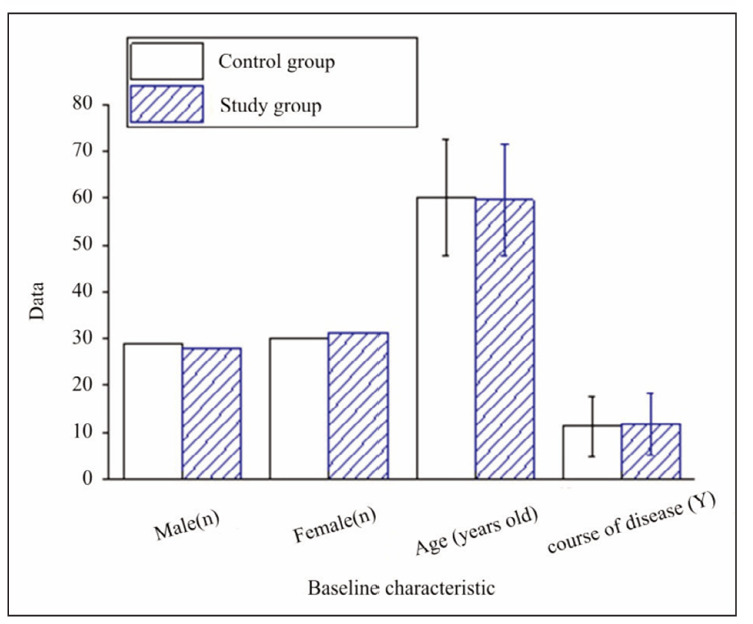
Contrast of general data of sick persons.

There were 29 men and 30 women in the controls, with a mean age of (60.24±12.53) and a disease course of (11.21±6.47) years. There were 28 men and 31 women in the study group, with a mean age of (59.58±11.76) and a disease duration of (11.62±6.53) years. There were no apparent distinctions in baseline characteristics of the patients with CHD (Figure 1).

### Cardiac function indexes of patients with CHD before and after treatment

There was no apparent distinction in cardiac function before and after intervention (*P*>0.05). Through treatment, LVESD and LVEDD of the controls and the study subjects were lower, and LVEF, CI, and SI were higher than before treatment. In addition, the LVESD, LVEDD, LVEF, CI, and SI of the study subjects were better than the controls (*P*<0.05), as shown in Table 2.

**Table 2:** Cardiac Function Indexes of Patients with CHD Before and After Treatment. *P<0.05 compared to before treatment. LVESD, left ventricular end-systolic diameter; LVEDD, left ventricular end-diastolic diameter; LVEF, left ventricular ejection fraction; CI, cardiac index; SI, systolic index.

Index		Control Group (n=59)	Study Group (n=59)	P
LVESD (mm)	Before	34.21±4.51	35.19±4.83	>0.05
	After	30.15± 3.92*	28.59±3.51*	<0.05
LVEDD (mm)	Before	54.38±5.62	55.63±5.91	>0.05
	After	49.23±4.92*	47.19±4.31*	<0.05
LVEF (%)	Before	55.12±8.31	53.49±9.15	>0.05
	After	62.19±7.51*	65.38±6.92*	<0.05
CI (L/min/m^2^)	Before	2.53±0.43	2.41±0.51	>0.05
	After	2.83±0.41*	3.02±0.39*	<0.05
SI (m/s)	Before	0.34±0.06	0.32±0.07	>0.05
	After	0.41±0.05*	0.45±0.04*	>0.05

### Incidence of angina pectoris in patients with CHD before and following treatment

There was no apparent distinction in the frequency and duration of angina pectoris and 6MWD in patients with CHD before remedy (*P*>0.05). Following the remedy, the frequency and duration of angina pectoris in the controls and the study subjects were lower than before (*P*<0.05), and the study subjects decreased more clearly. The 6MWD of study subjects was clearly increased relative to before remedy (*P*<0.05), and the increase of study subjects was more visible (Table 3).

**Table 3:** Changes in Angina Frequency, Exercise Tolerance, and Serum Inflammatory Markers after Pue Treatment.

Variable		Before<br>Treatment	After<br>Treatment	P-value<br>(Before vs After)	P-value<br>(Study vs Control)
Frequency of Angina Pectoris <br>(times/week)	Control	3.2±1.5	1.8±0.8	<0.05	<0.05
	Study	3.5±1.4	1.1±0.5		
Duration of Angina Pectoris <br>(min/episode)	Control	10.5±3.2	6.2±2.1	<0.05	<0.05
	Study	11.2±2.8	4.5±1.8		
6MWD (meters)	Control	420±50	480±40	<0.05	<0.05
	Study	410±60	520±30		
Serum NLRP3 inflammasome<br>(ng/mL)	Control	1.5± 0.8	0.8±0.4	<0.05	<0.05
	Study	1.8±0.6	0.5±0.3		
Serum CTRP3 (ng/mL)	Control	102.5±2.0	124.5±2.5	<0.05	<0.05
	Study	90.5±1.5	150.2±2.2		
Serum Lp-PLA2 (ng/mL)	Control	148.8±58.4	120.3±47.5	<0.05	<0.05
	Study	187.1±60.6	134.9±56.2		

There was no apparent distinction in serum NLRP3 inflammasome, CTRP3, and Lp-PLA2 levels in patients with CHD before and following the remedy. Following the remedy, the serum NLRP3 inflammasome and Lp-PLA2 in the controls and the study subjects were clearly lower than before the remedy (*P*<0.05), and the improvement in the study subjects was more obvious. The serum CTRP3 of the controls and the study subjects was clearly higher as against before remedy (*P*<0.05), and the improvement of the study subjects was more evident (Table 3).

### Adverse reactions and total effectiveness in patients with CHD

In the controls, there were 1 case of nausea and vomiting, 3 cases of abdominal distension, 3 cases of rash, and 2 cases of drowsiness. There were 2 cases of nausea and vomiting, 1 case of abdominal distension, 1 case of rash, and 3 cases of drowsiness in the study subjects. The incidence of adverse reactions in the study subjects was lower than against the controls (11.86% vs 15.25%, *P*>0.05).

The total effective rate of the study subjects was clearly superior (93.22% vs 81.36%, *P*<0.05).

## Discussion

Our study discovered that puerarin administration reduced serum lipoprotein-associated phospholipase 2 (Lp-PLA2) levels and improved lipid metabolism in elderly patients with CHD, while another study found that short-term puerarin supplementation did not improve lipid profiles in healthy Chinese men [19]. There are several potential reasons for the differences in findings between the two studies, such that the mentioned study included healthy Chinese men aged 18–50. In contrast, our study focused on elderly patients with CHD.

A randomised controlled trial by Yang et al. [20] found that 24 weeks of treatment with 400 mg of puerarin significantly reduced carotid intima-media thickness (CIMT) in patients with active rheumatoid arthritis (RA), which may be associated with improved insulin resistance. While our and Yang et al.’s [20] studies differed in population and outcome measures, they both suggest that puerarin may benefit cardiovascular health, warranting further investigation.

Similar to our study, in a study by Shi et al. [21], puerarin treatment for 3 weeks improved insulin resistance, lipid profiles, and fibrinolytic activity in patients with CHD. Specifically, puerarin decreased fasting plasma insulin (FINS) and improved insulin sensitivity index (ISI) while also lowering total cholesterol, triglyceride, and LDL-C levels and increasing HDL-C and tissue plasminogen activator (tPA) activity.

Another randomised, double-blind, placebocontrolled, two-way crossover trial by Kwok et al. [22] found that puerarin supplementation for 12 weeks reduced fasting glucose and improved lipid profiles in Chinese men. In contrast, our study found that puerarin administration decreased serum NOD-like receptor protein 3 (NLRP3) inflammasome and lipoprotein-associated phospholipase 2 (Lp-PLA2) levels while increasing C1q/TNF-Related Protein 3 (CTRP3) levels in elderly patients with coronary heart disease (CHD). Both studies suggest that puerarin may benefit cardiovascular health by improving lipid metabolism and reducing inflammation. However, the two studies have some differences in the study design and population. Kwok et al. [22] included Chinese men aged 18–50 years, while our study included elderly patients with CHD. The study by Kwok et al. [22] had a larger sample size (n=217) than ours (n=59). Additionally, the study by Kwok et al. [22] had a more rigorous study design, with a randomised, double-blind, placebo-controlled, two-way crossover trial. In contrast, our study had a retrospective cohort study design.

Pue has an inhibitory outcome on inflammation, which can reduce the inflammatory response in coronary atherosclerotic plaques, suppress leukocyte adhesion and the release of inflammatory mediators, and reduce the progression of plaques [23]. Pue has been found to suppress the production of malondialdehyde (MDA) and 4-hydroxynonenal (4-HNE), decrease the expression of prostaglandin endoperoxide synthase (Ptgs)2 mRNA, and increase the expression of glutathione peroxidase 4 (GPX4) protein in myocardial ischemia/reperfusion (I/R) mice. Pue has protective outcomes on myocardial I/R injury by suppressing ferroptosis and inflammation [24]. It was found that Pue adjuvant remedy could reduce the serum expression of NLRP3 inflammasome in patients with CHD, and the serum level of NLRP3 inflammasome in PUE-treated rats was also markedly lower as against CHD rats, indicating that Pue reduced the release of inflammatory mediators and the degree of inflammatory response by regulating the NLRP3 inflammasome signalling pathway. These results suggested that PUE could alleviate the symptoms of CHD. The serum CTRP3 of the study subjects was markedly higher than that of the controls following the remedy, and the serum CTRP3 of the Pue-treated rats was markedly higher than that of the CHD rats. CTRP3 is associated with inflammation and metabolism and plays an essential role in CHD. Pue can markedly increase the expression of CTRP3, thereby suppressing inflammatory response, improving vascular function, and reducing plaque formation. The lower the CTRP3 level is, the higher the severity of CHD is. Pue also suppressed oxidative stress and lipid peroxidation and reduced lipid deposition. Some studies have shown that Pue can suppress the activation of the Wnt signalling pathway, reduce the phosphorylation of p65 through the crosstalk between Wnt and NF-κB signalling pathways, and alleviate isoproterenol-induced cardiac hypertrophy. Pue also has anti-platelet aggregation and anticoagulant outcomes, which can reduce plasma fibrinogen levels and reduce the risk of platelet activation and thrombosis. The serum Lp-PLA2 of the study subjects in this article was markedly lower than that of the control subjects following the remedy, and the serum Lp-PLA2 of the PUE-treated rats was considerably lower than in CHD rats. The increased plasma Lp-PLA2 indicates an increased risk of restenosis and adverse cardiovascular events, and its expression is increased in coronary atherosclerotic plaques. These results suggest that Pue can reduce the risk of cardiovascular events by down-regulating the expression and activity of Lp-PLA2 and reducing plaque instability.

The results revealed that the Pue remedy had a visible clinical outcome in patients with CHD. Patients treated with Pue had visible improvements in cardiac function measures, angina episodes, resting electrocardiogram, cardiac insufficiency, and serum inflammatory measures as against controls who did not receive Pue. Pue can markedly reduce the frequency and duration of angina attacks and improve exercise tolerance during angina attacks. In addition, Pue also reduced serum NLRP3 inflammasome and Lp-PLA2 and increased CTRP3. The results indicate that Pue remedy can improve cardiac function, slow down angina attacks, and regulate the inflammatory response in patients with CHD, so it has important clinical application value. Although the study subjects had a small reduction in the incidence of adverse outcomes, the difference was not visible. Overall, this trial provides a new and practical option for the remedy of CHD and provides the basis for further relevant research.

## Conclusion

The study’s findings suggest that puerarin may be a helpful adjunct therapy in managing coronary heart disease, particularly in elderly patients. The reduction in angina episodes and improvement in exercise tolerance observed in the puerarin-treated group compared to the control group suggests that puerarin may positively impact cardiovascular health. The study’s findings also provide mechanistic insights into puerarin’s potential anti-inflammatory effects and may have implications for lipid metabolism. While the study’s findings are promising, several limitations must be acknowledged. The study was retrospective and had a relatively small sample size, which may limit the generalizability of the results.

## Dodatak

### Fundings

The research is supported by the Industry Research Special Fund Project of the State Administration of Traditional Chinese Medicine (No. 201507027), Tang Qisheng Qihuang Scholar Program of the State Administration of Traditional Chinese Medicine (No. 209-137), supported by the National Natural Science Foundation of China (No. 81473658).

### Conflict of interest statement

All the authors declare that they have no conflict of interest in this work.
